# Fast sorption measurements of volatile organic compounds on building materials: Part 1 – Methodology developed for field applications

**DOI:** 10.1016/j.dib.2016.01.011

**Published:** 2016-01-13

**Authors:** M. Rizk, M. Verriele, S. Dusanter, C. Schoemaecker, S. Le Calve, N. Locoge

**Affiliations:** a Mines Douai, SAGE, F-59508 Douai, France; b Université de Lille, F-59000 Lille, France; c Université de Lille 1, Laboratoire de Physico-chimie des Processus de Combustion et de l׳Atmosphère, Villeneuve d׳Ascq, France; d Université de Strasbourg/CNRS, Institut de Chimie et Procédés pour l’Energie, l’Environnement et la Santé (ICPEES), UMR 7515, F-67087 Strasbourg, France

## Abstract

A Proton Transfer Reaction-Mass Spectrometer (PTR-MS) has been coupled to the outlet of a Field and Laboratory Emission Cell (FLEC), to measure volatile organic compounds (VOC) concentration during a sorption experiments (Rizk et al., this issue) [1]. The limits of detection of the PTR-MS for three VOCs are presented for different time resolution (2, 10 and 20 s). The mass transfer coefficient was calculated in the FLEC cavity for the different flow rates. The concentration profile obtained from a sorption experiment performed on a gypsum board and a vinyl flooring are also presented in comparison with the profile obtained for a Pyrex glass used as a material that do not present any sorption behavior (no sink). Finally, the correlation between the concentration of VOCs adsorbed on the surface of the gypsum board at equilibrium (*C_se_*) and the concentration of VOCs *C_e_* measured in the gas phase at equilibrium is presented for benzene, C8 aromatics and toluene.

## **Specifications Table**

**Table:** 

Subject area	*Chemistry*
More specific subject area	*Indoor air quality*
Type of data	*Table, graph*
How data was acquired	*PTR-ToFMS (Kore technology)*
Data format	*Analyzed*
Experimental factors	*50*±*5% at 23*±*2* *°C*
Experimental features	*Very brief experimental description*
Data source location	–
Data accessibility	–

## Value of the data

• This data is important for readers that want to use the FLEC-PTRMS method for fast sorption experiments.
• The limits of detection of the PTR-MS are important to validate the PTR-MS performance and to compare this performance with other analytical device.
• The concentration profiles obtained for the first time using FLEC-PTRMS method can be compared with literature data obtained used larger experimental chamber coupled to gas chromatography analyzer.

## 1 Data

Sorption rates of volatile organic compounds (VOCs) onto/from indoor surfaces are key parameters driving their indoor concentrations. Several models have been developed to simulate the VOC emission/sorption by indoor surfaces taking into account different key parameters such as the sorption coefficients, the diffusion coefficients and the mass transfer coefficient through the boundary layer. Some of the models are presented in this article. The experimental method developed to perform fast measurements of VOC sorption parameters on the field by coupling a Field and Laboratory Emission Cell (FLEC) to a Proton Transfer Reaction-Mass Spectrometer (PTR-MS), is also presented.

## 2 Experimental design, materials and methods

Several models have been developed to simulate the VOC emission/sorption by indoor surfaces. Table 1 presents a comparison between some of the typical models used in literature.

The setup used is based on coupling a FLEC (Chematec) and a high resolution PTR-MS (PTR-ToFMS, Kore technology) [1]. The FLEC inlet is connected to two gas generation systems using a three-way valve. The first generation system is composed of a dry zero air generator (Claind) and a humidificator made of a water bubbler and mass flow controllers (MKS). This system is used to supply the FLEC with humid clean air at constant flow rate (200–500 mL min^−1^) and stable relative humidity (50±5% at 23±2 °C). The second generation system is made of a VOC cylinder connected to a dilution system (Gas Calibration Units-Ionicon Analytik), which is used to dilute the VOC mixture at a constant relative humidity of 50%. The FLEC’s outlet is connected to the PTR-MS to quantify VOC concentrations exiting the cell. An exhaust is left at atmospheric pressure to prevent a pressure build-up in the FLEC apparatus.

First of all, a blank experiment is carried out on a Pyrex glass before each sorption experiment on a tested material and using the same procedure. This experiment, referred as “no sink” in the following, allows evaluating sorption processes on internal surfaces of the FLEC apparatus and the Teflon tubing. A sorption experiment involves a 3-step procedure as described in the following. The FLEC is first exposed on a material and supplied with humidified zero air. When concentrations reach relatively steady state, the FLEC is supplied with humid air containing targeted VOCs. This second phase is named “adsorption phase”. During this step, VOCs concentrations increase until an equilibrium is reached where the concentrations are equal to those registered for the adsorption phase already performed on the Pyrex glass. Once the VOCs concentrations are stable, humidified zero air is provided to the cell instead of the VOCs mixture. This third phase is named “desorption phase”. During this step, VOC concentrations decrease until steady concentrations similar to those reached in the end of the first phase, are observed. Standard operating conditions were defined as an air temperature of 23±2 °C and a relative humidity of 50±5% for all experiments described in [1].

**Fig. 1:**
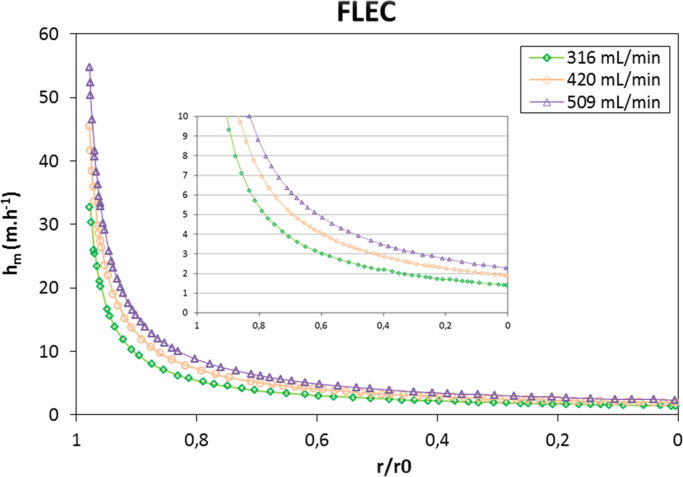
The mass transfer coefficient along the FLEC radius calculated from the local Sherwood number as presented in [5].

The VOCs background signals measured with PTR-MS using zero air were used to estimate detection limits (LOD) as three times the standard deviation on the zero measurements. Measured LODs (Table 1) are less than 3.4, 4.7 and 11 µg m^−3^ for a time resolution of 20, 10 and 2 s respectively and which is low enough to measure accurately the concentrations used in [1] (Fig. 1).

Zhang [5] studied the flow filed in the FLEC cavity and calculated the local Sherwood number *Sh_L_* for the different flow rates (186–509 mL min^−1^). According to this work, the mass transfer coefficient was calculated in the FLEC cavity for the different flow rates varying between 300 and 500 mL min^−1^. The equations used are presented in the following:

(1) $$Sh_{L}=0.3359R_{e}S_{c}{\left(\frac{r_{0}-r}{2\delta }\right)}^{-0.834}$$

(2) $$R_{e}=\frac{F_{v}}{\upsilon \pi r_{0}}$$

(3) $$S_{c}=\frac{2h_{m}\delta }{D_{v}}$$

where *Sh_L_* is the local Sherwood number, *R_e_* is the Reynolds number, *S_c_* is the Schmidt number, *r*_0_ is the maximum radius of the tested surface using the FLEC (m), *r* is the radiate coordinate (m), *δ* is the spacing between the emission surface (m), *F_v_* is the volumetric air flow rate (m^3^ h^−1^), *v* is the kinematic viscocity of air (m^2^ s^−1^), *h_m_* is the mass transfer coefficient (m h^−1^) and *d_v_* is the vapor diffusivity in air mixture (m^2^ s^−1^).

**Fig. 2:**
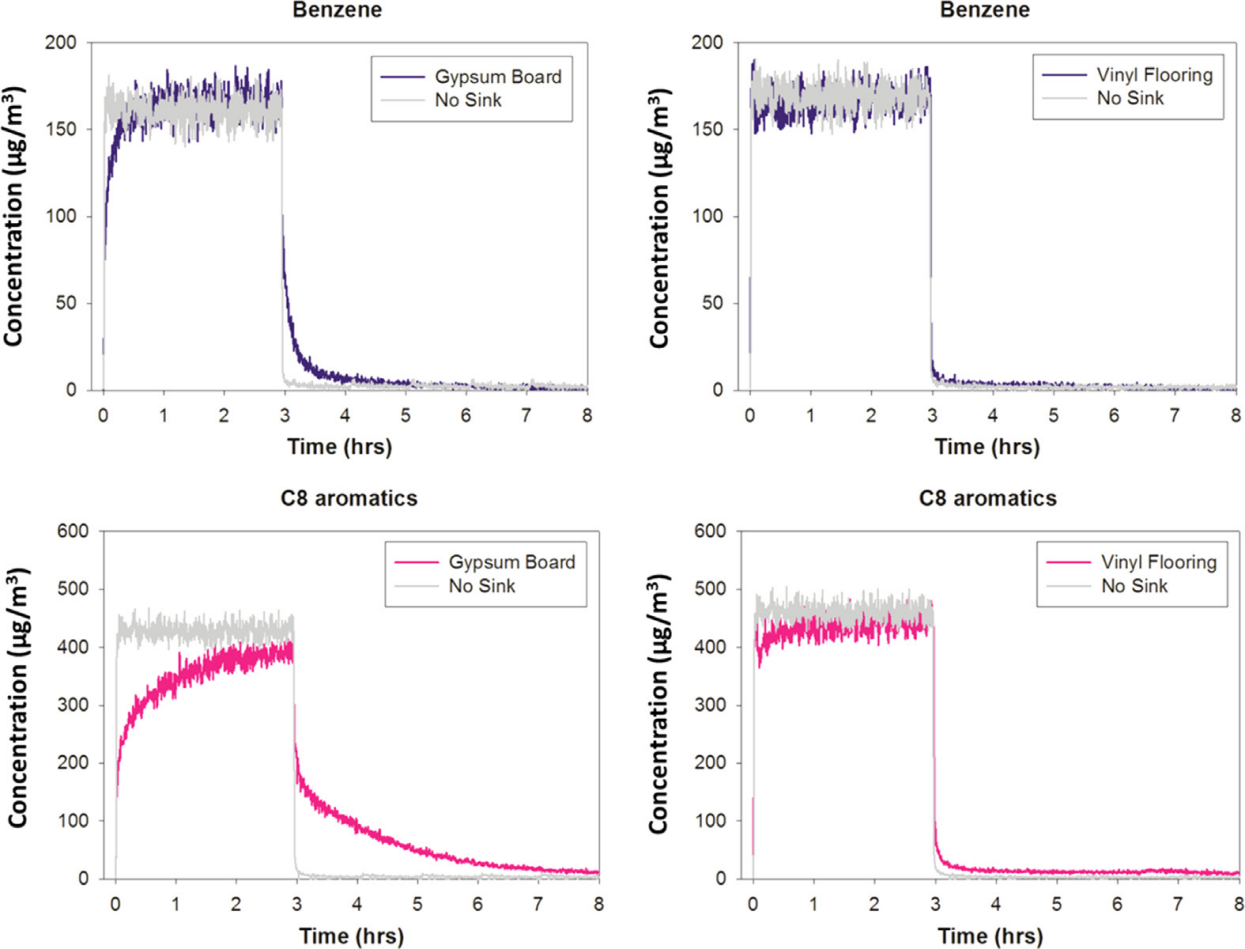
Concentration profiles measured during sorption experiments on a gypsum board and a piece of vinyl flooring for benzene and C8 aromatics. The “no sink” curve is also shown.

Time-resolved concentration profiles obtained for benzene and C8 aromatics are shown in Fig. 2 for two experiments performed on a gypsum board and a piece of vinyl flooring in comparison with the profiles obtained using a Pyrex glass which not present any sorption effect (no sink) (Table 2).

**Fig. 3:**
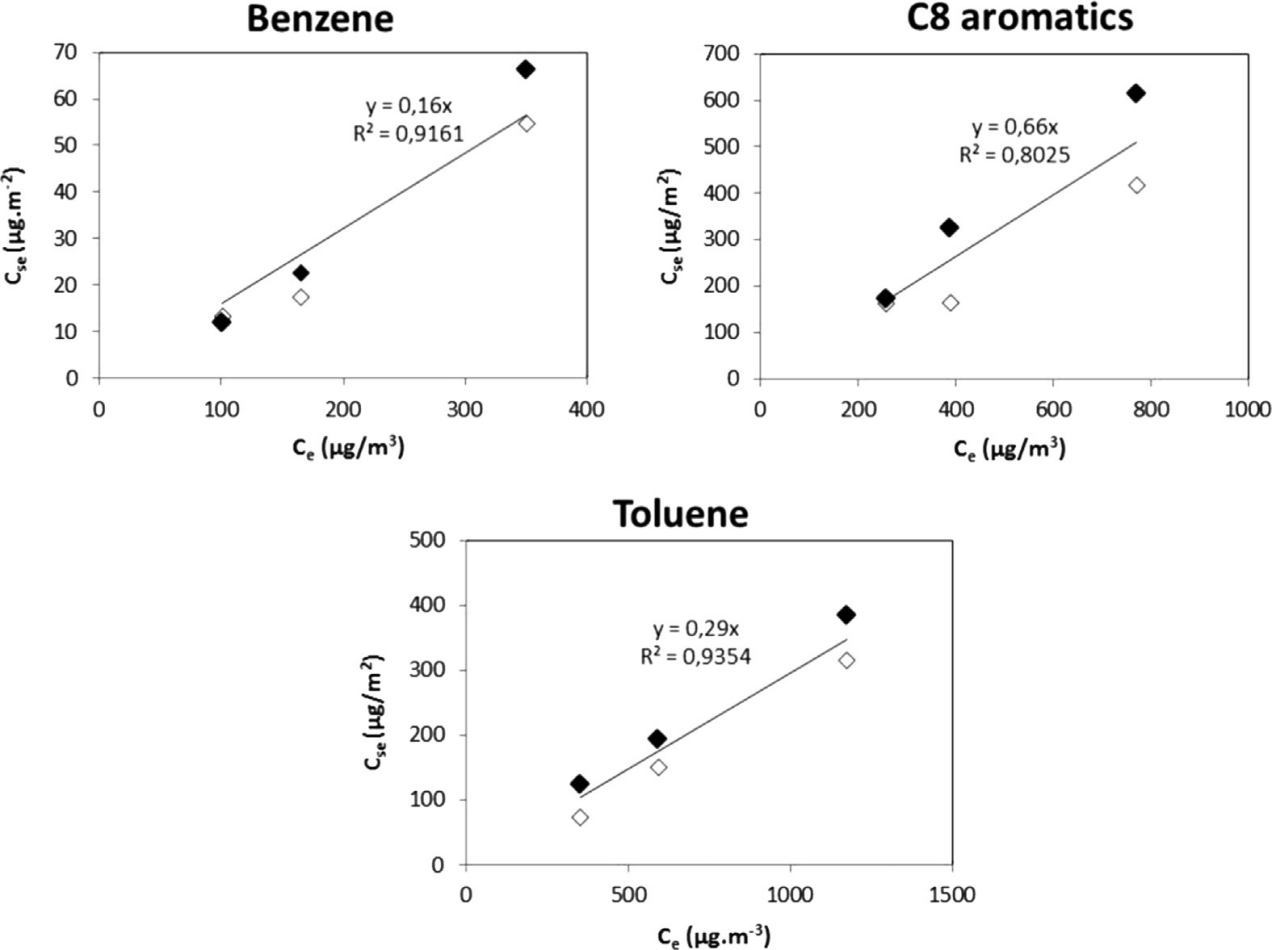
Correlation between *C_se_* and *C_e_* measured for the gypsum board with benzene, C8 aromatics and toluene. Full diamonds represent the adsorption phase and empty ones represent the desorption phase.

The applicability of the Langmuir isotherm was verified by investigating the partitioning of VOCs between the gas and adsorbed phases when the equilibrium is reached. The relationship between the concentration of VOCs adsorbed on the surface of the gypsum board at equilibrium (*C_se_*) and the concentration of VOCs *C_e_* measured in the gas phase at equilibrium (experimental measurements [1]) for the gypsum board and target VOCs, shows a linear trend with a correlation coefficient of 0.92, 0.80 and 0.94 respectively for benzene, C8 aromatics and toluene (Fig. 3). According to the relation (*C_se_*=*C_e_*×*K_e_*), the slope of the regression line should be equal to *K_e_*. The slope determined for benzene (0.16), C8 aromatics (0.66) and toluene (0.29) is in excellent agreement with the average *K_e_* value derived from 15 measurements [1]. This linear relationship confirms that Langmuir equilibrium can be applied at the concentrations used in [1] (106–1131 µg m^−3^). Moreover, these plots show the reversibility of the sorption phenomena. However, for the C8 aromatics the mass of desorbed compounds is underestimated due to a technical problem in the acquisition of the end of the desorption phase on the no-sink curve. The experiment done on the lower concentration show the reversibility of the phenomena on these compounds.

## Figures and Tables

**Table 1:** Comparison of some typical VOC emission/sorption mathematical models.

**Model equations**	**Parameters**	**Surface sorption**	**Diffusion inside material**	**Mass transfer through the boundary layer**	**Reference**
$\frac{dC_{g}}{dt}=NC_{in}-NC_{g}-k_{a}C_{g}L+k_{d}C_{s}L$	*k_a_*, *k_d_*	+	−	−	[2]
$\frac{\partial C_{m}(x,t)}{\partial t}=D_{m}\frac{\partial^{2}C_{m}(x,t)}{\partial x^{2}}$	*D_m_*, *K_p_*	+	+	−	[3]
$C_{m}(0,t)=KC^{*}(t)$
$C_{m}(L,t)=0$
$-D_{m}\frac{\partial C_{m}(x,t)}{\partial x}=h_{m}\left(y_{0}\left(t\right)-y\left(t\right)\right)$	*D_m_*, *K*_*p*,_*h_m_*	+	+	+	[4]
$V\frac{dy\left(t\right)}{dt}=Ah_{m}\left(y_{0}\left(t\right)-y\left(t\right)\right)-Qy\left(t\right)$
$K=\frac{{C\left(x,t\right)|}_{x=L}}{y_{0}\left(t\right)}$

**Table 2:** Limits of detection (3*σ*) for the 3 targeted VOCs measured with PTR-MS.

**PTR-MS time resolution**	**LOD (µg m**^**−3**^**)**		
**Benzene (*****m*****/*****z*****=79)**	**C**_**8**_**aromatics (*****m*****/*****z*****=107)**	**Toluene (*****m*****/*****z*****=93)**
20 s	1.0	2.3	3.4
10 s	1.3	3.2	4.7
2 s	3.0	7.0	11
